# Performance Evaluation of a Commercial Real-Time PCR Assay and of an In-House Real-Time PCR for *Trypanosoma cruzi* DNA Detection in a Tropical Medicine Reference Center, Northern Italy

**DOI:** 10.3390/microorganisms8111692

**Published:** 2020-10-30

**Authors:** Silvia Stefania Longoni, Elena Pomari, Alberto Antonelli, Fabio Formenti, Ronaldo Silva, Stefano Tais, Salvatore Scarso, Gian Maria Rossolini, Andrea Angheben, Francesca Perandin

**Affiliations:** 1 Department of Infectious-Tropical Diseases and Microbiology, IRCCS SacroCuore Don Calabria Hospital, Negrar di Valpolicella, 37024 Verona, Italy; elena.pomari@sacrocuore.it (E.P.); fabio.formenti@sacrocuore.it (F.F.); ronaldo.silva@sacrocuore.it (R.S.); stefano.tais@sacrocuore.it (S.T.); salvatore.scarso@sacrocuore.it (S.S.); andrea.angheben@sacrocuore.it (A.A.); francesca.perandin@sacrocuore.it (F.P.); 2 Department of Experimental and Clinical Medicine, University of Florence, 50134 Florence, Italy; alberto.antonelli@unifi.it (A.A.); gianmaria.rossolini@unifi.it (G.M.R.); 3 Microbiology and Virology Unit, Careggi University Hospital, 50134 Florence, Italy

**Keywords:** Chagas disease, *Trypanosoma cruzi*, real-time PCR, DNA

## Abstract

Chagas disease, a neglected protozoal disease endemic in Latin America, is also currently considered an emerging threat in nonendemic areas because of population movements. The detection of *Trypanosoma cruzi* DNA is increasingly being considered as important evidence to support Chagas disease diagnoses. However, further performance evaluation of molecular assays is useful for a standardization of strategy considering the whole process in routine diagnosis, especially for the different settings such as endemic and nonendemic countries. Seventy-five samples were collected from subjects screened for Chagas disease in Italy. The DNA was isolated from blood using automated extraction. We evaluated the performance of the commercial RealCycler^®^ CHAG kit (pmPCR) based on satellite DNA (SatDNA) and of an in-house real-time PCR (ihPCR) targeting Sat and kinetoplast (k) DNAs, using the concordance of two serology assays as a reference standard. The sensitivity of kDNA and SatDNA tests by ihPCR and SatDNA by pmPCR were 14.29% (95% confidence interval (CI) 6.38 to 26.22), 7.14% (95% CI 1.98 to 17.29), and 7.14% (95% CI 1.98 to 17.29), respectively. Specificity was 100% for all PCR assays and targets. Overall, our results suggest that the preferred approach for clinical laboratories is to combine the kDNA and SatDNA as targets in order to minimize false-negative results increasing sensitivity.

## 1. Introduction

Chagas disease had been described for the first time in 1909 by the Brazilian medical doctor Carlos Chagas. It is a neglected tropical disease (NTD) caused by the protozoa, single-celled eukaryotes, haemoflagellate *Trypanosoma cruzi*. It is transmitted mainly by a bed-bug insect called “kissing bugs” or “blood suckers” in endemic areas, a triatominae vector *Triatoma* spp., a subfamily of the Reduviidae [1]. Chagas disease is endemic in the Americas from the South of Texas to the North of Argentina [2]. *T. cruzi* infection is characterized by an acute phase followed by a chronic phase. During the acute stage, the parasitemia is usually detectable but symptoms are frequently absent or unspecific [2]. Following the acute stage, which lasts about 2 months, the disease enters into a chronic phase, which could persist for several decades or indefinitely without any organ involvement. The asymptomatic chronic stage is called “chronic indeterminate Chagas disease,” and it is characterized by very low parasitemia, frequently undetectable and interpreted as a fluctuant parasitic load [2,3]. Only ~30% of the infected population will develop cardiac and/or gastrointestinal disease, this symptomatic chronic stage is called “chronic determinate Chagas disease,” and it leads to morbidity or death if untreated [4]. The disability-adjusted life years (DALYs) caused by Chagas disease, globally is up to 10.8 [5,6].

The epidemiology of Chagas disease has changed as a consequence of population movements with an increasing number of infections also encountered in nonendemic areas, such as Australia, Japan, and Europe [7,8]. Among the European countries, Spain, the United Kingdom, France, Switzerland, and Italy host the most migrants from areas where Chagas disease is endemic, and Bolivians represent the highest rate of infected subjects [9]. The congenital route of transmission currently represents the main transmission mode of *T. cruzi* in nonendemic areas, but a serious threat of infection is also represented by blood transfusion and organ transplantation in nonendemic areas [2,3,10,11,12]. Only few studies up until now have reported the prevalence of Chagas disease among migrants from endemic countries, and most of them present a bias due to selection criteria. Indeed, the real prevalence of Chagas disease in Europe is not clear [9]. About the congenital route, it is estimated as an average of 6% ranging from 2.6 to 13.8% of transmission, whereas the estimated rate of prevalence of Chagas disease transmission during blood transfusion, organs transplantation, or the iatrogenic route is even lower ranging from 0.007 to 7.7 [9]. Unfortunately, the real numbers of the epidemiology of Chagas disease in Europe are far from the reality. This is due to the underestimation of the number of migrants, the lack of a proper surveillance protocol, and the lack of high-quality epidemiological studies for this disease [8,9,13].

The diagnosis for the acute stage of Chagas disease, as well as in the case of reactivation of the disease during immunosuppression, is commonly done by microscopic observation of the parasite (mainly through the microhematocrit technique or Strout’s method). For the diagnosis of chronic Chagas disease, in which the parasitemia is low and intermittent, serological methods are recommended by the World Health Organization (WHO) [14] and are largely used. Instead, parasitemia detection is difficult even with molecular techniques such as polymerase chain reaction (PCR), due to the scarce presence of bloodstream-circulating parasites in the chronic stage. Currently the diagnosis of chronic Chagas disease is based, according to the WHO, on the detection of antibodies against *T. cruzi* using two different serological tests based on distinct antigens [2,15]. For all these reasons, it is always more necessary to find a common strategy for the evaluation of an at-risk population for chronic Chagas diseases in nonendemic realities.

Molecular approaches for the detection of *T. cruzi* DNA by PCR have been developed across time, with a wide range of sensitivities (45–96.5%) due to different factors, such as the genetic variability among different *T. cruzi* and also the PCR strategies used (e.g., volume and storage of samples, DNA extraction, target sequence) [4,16,17,18]. In particular, the evaluation of different PCR tests showed poor sensitivity for diagnostic purposes of chronic disease [19]. In 2017, it was demonstrated that automated extraction of the DNA improved the quality of the PCR [20]; nevertheless, a common protocol is not available yet [4,21]. Nowadays, the in-house techniques are commonly used in the reference sites in endemic and nonendemic countries [22,23,24]. One of the most used genetic targets is the nuclear satellite DNA (SatDNA), a 195-nucleotide sequence repeated about 10^5^ times in the *T. cruzi* nuclear genome [20], representing 10% of the total cellular DNA [25]. Additionally to SatDNA, the variable region of the kinetoplast (kDNA) minicircle has been proposed as a PCR target, achieving efficient *T. cruzi* DNA detection in patients with chronic Chagas disease by PCR [26]. *T. cruzi* mitochondrial DNA, so-called kinetoplast DNA, represents 15−30% of the total cellular DNA [27].

To date, there are few commercially available real-time PCR assays for the routine laboratory detection of *T. cruzi* DNA in clinical samples. In the present study, we intend to describe the performances of an in-house real-time PCR (ihPCR) based on SatDNA and kDNA, developed and validated by Ramirez and co-workers [28], and of the commercial RealCycler^®^ CHAG kit (pmPCR) based on SatDNA targets for the detection of *T. cruzi* DNA. For this purpose, we retrospectively collect samples of subjects with known diagnoses of Chagas disease determined by serology as recommended by the WHO [14]. We evaluated the performance of the in-house and commercial PCRs after the automated DNA isolation as the most performant technique for the nucleic acid extraction [29], and according to our diagnostic routine. 

## 2. Materials and Methods 

**Figure 1:**
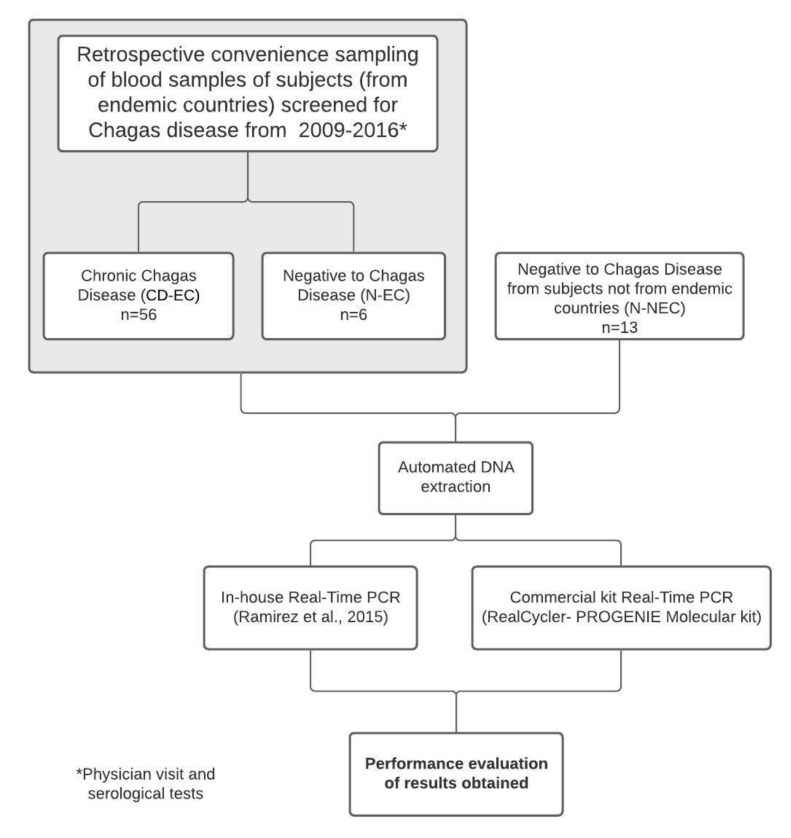
Study flowchart. CD-EC: Chronic Chagas disease from endemic country; N-EC: Negative from endemic country; N-NEC: Negative from nonendemic country.

In this study, we considered data obtained from two real-time PCR methods, an in-house real-time PCR [28] and a commercial kit, RealCycler^®^ CHAG kit (Progenie Molecular, Valencia, Spain), performed on DNA automatically extracted from blood samples of patients diagnosed with chronic Chagas disease. The flow of this study is described in Figure 1. 

### 2.1. Description of the Population

We included a total of 75 samples retrospectively collected. Among these, 62 samples were from subjects screened for Chagas disease between 2009 and 2016 during activities of information and education about Chagas disease across the Latin American community (mainly composed by Bolivians) living in the Bergamo and Verona Provinces (respectively Lombardy and Veneto region, Northern Italy). Positivity for Chagas disease was defined by at least two serology tests, as recommended by the WHO [14] and by the clinical examination. Sample distribution was as follows: 56 chronic chagasic individuals from endemic countries (CD-EC), 6 nonchagasic individuals from endemic countries (N-EC), and 13 nonchagasic individuals from nonendemic countries (N-NEC). All the serum samples were tested with two different serology tests among the following three: One enzyme-linked immunosorbent assay (ELISA) based on a recombinant antigen (Biokit S.A., Lliçàd’Amunt, Spain), one ELISA based on whole-parasite lysate (Wiener Laboratorios, Rosario, Argentina), and an immunocromatographic test (Cypress Diagnostic, Langdorp, Belgium). We performed all the tests according to the manufacturer’s recommendations as detailed below. Patients with Chagas disease were examined following an internal protocol that included hematology and biochemistry tests, as described in our previous study [30]. A summary of the subjects’ characteristics is displayed in Table 1 and Table S1.

### 2.2. Ethical Statement

In accordance with the Helsinki Declaration of 1975, revised in 1983, this study was submitted for its evaluation to the competent Ethics Committee (Comitato Etico for Clinical Research of Verona and Rovigo Provinces). The Ethics Committee approved this study (no. 11330) on 20 February 2019. All included patients signed an informed consent form for the donation of their biological samples for research purposes at our center. All the research was performed in accordance with the relevant guidelines/regulations.

### 2.3. Blood Collection

For the serological survey, peripheral blood was collected by venipuncture into an EDTA (ethylenediaminetetraacetic acid)-free tube from each patient and control subject. After an incubation period of 30 min at room temperature, the tubes were centrifuged in order to remove the clot at 1750× *g* and then the sera collected, and they were immediately used to perform the serology tests or stored at −80 °C until their use.

For real-time PCR, EDTA peripheral blood was collected from each patient and control subject and immediately mixed with an equal volume of 6 M guanidine hydrochloride/0.2M EDTA solution (pH 8.00), as previously described [31]. After 24 h at room temperature, samples were stored at 4 °C until automated DNA isolation. 

### 2.4. Serology Assay

Three commercial serological assay kits were used to screen serum samples: Two ELISAs and one rapid diagnostic test (RDT), as described below. 

#### 2.4.1. BioELISA Chagas (Biokit S.A., Lliçàd’Amunt, Spain)

This commercial kit is based on a recombinant antigen that is coated to the bottom of microtiter wells. Following the manufacturer’s instructions, each serum sample at a final dilution of 1/21 (10 µL serum/200 µL sample diluent) was added to each corresponding well. The anti-human IgG and anti-human IgM conjugate, labeled with horseradish peroxidase, was provided within the kit. An internal negative, as well an internal positive, control was included in the kit. The cut-off value was calculated for each plate as the following: The mean of the optical density (OD) of three negative replicates plus fixed values of 0.300. The final results were obtained by normalizing the OD obtained from each serum sample and dividing them by the cut-off value (ODsample/ODcutoff). Data were categorized as positive if ≥1, negative if <0.9, and equivocal if ≥0.9 but <1.0.

#### 2.4.2. Chagastest ELISA Lisado (WienerLaboratorios, Rosario, Argentina)

This commercial kit is based on whole-parasite lysate; the antigens correspond to highly conserved areas between different strains. Following the manufacturer’s instructions, each serum sample at a final dilution of 1/11 (20 µL serum/200 µL sample diluent) was added to each corresponding well. The anti-human IgG conjugate, labeled with horseradish peroxidase, was provided within the kit. An internal negative, as well an internal positive, control was included in the kit. The cut-off value was calculated for each plate as the following: The mean of the OD of the two negative replicates plus a fixed value of 0.200. Data were categorized as positive if the sample OD was higher than the cut-off value and negative if the sample OD was lower than the cut-off value. In order to homogenize the results in the routine process, we normalized it by dividing the OD of the sample by the cut-off value (ODsample/ODcutoff). Data were categorized as positive if ≥1, negative if <0.9, and equivocal if ≥0.9 but <1.0.

#### 2.4.3. Chagas Quick TEST (Cypress Diagnostic, Langdorp, Belgium)

This commercial kit is a rapid immunochromatographic test based on a multi-epitope recombinant antigen. This rapid test is able to detect antibodies against *T. cruzi* either in serum (adding 10 µL) or blood (adding 20 µL). After adding the serum sample, following the manufacturer’s instructions, three drops of provided buffer were added and incubated for a period of 10 min. It presents a control line to validate the test. Positivity was given by the presence of a red test line together with the control line, while negativity was given by the absence of a red test line and with the control line. If the control line was not revealed, the test was discarded and repeated.

### 2.5. DNA Isolation

For automated DNA isolation, 200 µL of the whole blood-guanidine sample, as previously reported [20,23], was transferred to the cartridge sample for automatic extraction using a MagnaPureLC.2 instrument (Roche Diagnostics, Basilea Switzerland) following the protocol DNA_I_Blood_Cells_Highperformance_II and using the DNA isolation kit I (Roche) with a final elution volume of 100 µL. The DNA samples were kept at −20 °C until further molecular analysis.

### 2.6. Real-time PCR Analysis

The 75 DNA specimens were examined using the ihPCR assay and the commercial pmPCR assay, on the CFX96 system (Bio-Rad, Hercules, CA, USA) (Table 2). 

Briefly, the ihPCR assay consisted of two real-time PCRs (rt-PCRs) using two sets of primers and probes specific for SatDNA (Forward sequence 5′-ASTCGGCTGATCGTTTTCGA-3′; Reverse sequence 5′-AATTCCTCCAAGCAGCGGATA-3′ and Probe sequence FAM-5′-CACACACTGGACACCAA-3′-MGB) and kDNA (Forward sequence 5′-TTTGGGAGGGGCGTTCA-3′; Reverse sequence 5′-ATATTACACCAACCCCAATCGAA-3′ and Probe sequence HEX-5′-CAT[+C]TCA[+C]C[+C]GTA[+C]ATT-3′-LNA), as previously described and validated [28,32]. The amplification reaction was performed using a SsoAdvanced Universal Probes Supermix (Bio-Rad) in a final volume of 25 µL with 5 µL of DNA; the PCR cycle protocol consisted of 3 min at 95 °C, followed by 40 cycles at 95 °C for 15 s and 58 °C for 1 min, with a final step of 72 °C for 1 min. As a control for PCR inhibitors and amplification, the exogenous *Arabidopsis thaliana* DNA was amplified with the specific primers/probe set (Forward sequence 5′-ACCGTCATGGAACAGCACGTA-3′; Reverse sequence 5′-CTCCCGCAACAAACCCTATAAAT-3′ and Probe sequence Cy5.5 -5′-AGCATCTGTTCTTGAAGGT-3′-MGB), as previously described and validated [28,33]. The pmPCR assay was performed according to the manufacturer’s instructions. Briefly, the SatDNA sequence was amplified by a specific primers/probe set provided in the RealCycler^®^ CHAG kit (Progenie molecular, Valencia, Spain). The exact sequences of the primers and probes are not available from *Progenie molecular* as part of intellectual property rights. The PCR cycle protocol consisted of 15 min at 95 °C, followed by 40 cycles at 95 °C for 15 s, 60 °C for 30 s, and 72 °C for 30 s. The kit also provided a competitive internal control to evaluate the possible inhibition of reaction. For both PCR assays, each run contained one negative (no DNA) and one *T. cruzi* DNA positive control. A sample was considered valid when the internal control was amplified with a Cycle threshold (Ct) ≤ 35. Results were expressed as Ct values stratified into high (Ct < 30), moderate (30 ≤ Ct ≥ 35), low (Ct > 35) DNA load and negative (Ct ≥ 40 cycles or no amplification detected).

### 2.7. Statistical Analysis

The sample size of this study was determined by the available number of archived specimens. All collected data were summarized using descriptive statistics. Age was reported as the median (Mdn) and first and third quartile (IQR). Estimated parameters were reported with 95% confidence intervals (CI). The statistical significance level was fixed at 5%. Both statistical methods and plots were used to assess test performances and agreements. The concordance between kDNA and SatDNA was estimated by the overall, positive, and negative agreements. To assess the agreement, Cohen’s Kappa coefficient was used. Test results were presented in contingency tables of frequencies from which sensitivity, specificity, and accuracy were derived by comparing them to the known diagnosis (using serology as a reference test). Predictive values were not calculated, due to the retrospective nature of this study. Data analysis was performed using SAS software version 9.4 (SAS Institute, Inc., Cary, NC, USA) and graphs were made using GraphPad Prism version 8.0.2 (GraphPad Software, San Diego, CA, USA).

## 3. Results

**Figure 2:**
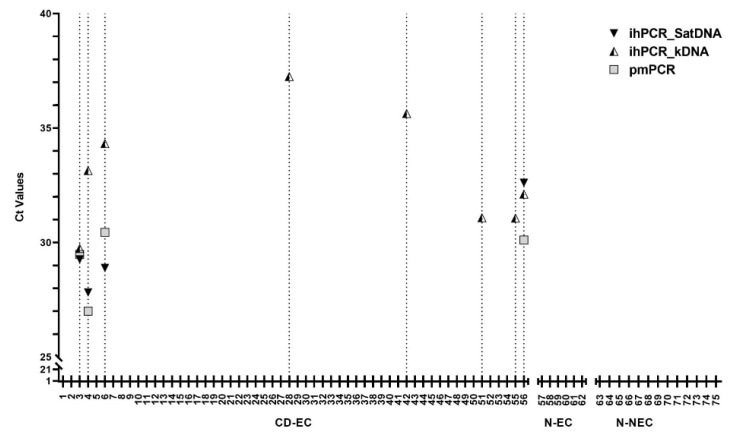
Ct values obtained with in-house real-time PCR (ihPCR) (satellite DNA (SatDNA) and kinetoplast DNA (kDNA)) and pmPCR. CD-EC = blood samples from patients with Chagas disease from endemic country; N-EC = blood samples from patients negative for Chagas disease from endemic country; N-NEC = blood samples from patients negative for Chagas disease from nonendemic country.

We analyzed a total of 75 blood samples of which 56 (74.67%) were from subjects with a known positive diagnosis for chronic Chagas disease. Our population included two different groups of negative control subjects coming from endemic and nonendemic countries, specifically 6 subjects from Bolivia and 13 subjects from Italy. All the negative control subjects gave negative results by the two serological tests (Table 1). In this study, an *A. thaliana* DNA sequence was used as an internal amplification control (IAC) in the ihPCR, as previously described by Duffy and co-workers [33], and it was detected in all 75 samples included in the study, suggesting no inhibition of reactions. Likewise, we observed the signal for the internal control included among the reagents supplied with the kit of the pmPCR. Concerning the *T. cruzi* DNA detection, of the 56 specimens classified as positive for Chagas disease by our routine diagnosis, 48 were negative with ihPCR to both SatDNA and kDNA targets (Cohen’s kappa of 0.6408, 95% CI 32.12–96.03), whereas 4 samples were positive to the SatDNA target by both PCRs (ihPCR and pmPCR), and 4 samples were positive only for the kDNA target by ihPCR (Figure 2).

The 19 specimens from patients diagnosed as negative by our routine diagnosis were confirmed as negative with both PCR assays (Figure 2). The sensitivities of kDNA and SatDNA tests by ihPCR (calculated based on clinical diagnosis) were 14.29% (95% CI 6.38 to 26.22) and 7.14% (95% CI 1.98 to 17.29), respectively. The sensitivity of the SatDNA test by pmPCR, calculated based on clinical diagnosis, was 7.14% (95% CI 1.98 to 17.29). The specificity was 100% for SatDNA and kDNA targets analyzed by both PCR tests (ihPCR and pmPCR) (Table 3).

The overall accuracy for the kDNA tested by ihPCR was 36% (CI 25.14–6.86) with 85.45% (CI 76.14–94.77) false negativity probability. For the SatDNA tested by ihPCR and pmPCR, we obtained the same overall accuracy of 30.67% (CI 20.23–41.10) and 92.73% false negativity probability (CI 85.86–99.59). The agreement of kDNA tested by ihPCR and SatDNA tested by both PCRs (ihPCR and pmPCR) was also assessed using Cohen’s kappa, resulting in 0.6411 (95% CI 0.3218 to 0.9605).

## 4. Discussion

The standardization of protocols for the diagnosis of Chagas disease is needed among laboratories. In particular, new strategies have been proposed with the introduction of commercial molecular tests. To the best of our knowledge, a few kits are commercially available for the routine laboratory detection of *T. cruzi* DNA in clinical samples in Italy. For this study, we chose the RealCycler^®^ CHAG Progenie Molecular PCR kit as available in our laboratory. The majority of the works in the literature assessed and evaluated in-house PCR-based assays, and they showed a very good performance in terms of sensitivity and specificity, especially in endemic areas. On the contrary, overall, our results obtained from an in-house PCR (ihPCR) and a commercial PCR (pmPCR) were similar to those obtained by previous international molecular studies for the detection of *T. cruzi* DNA in a nonendemic country monitoring cohorts of migrants from Latin America [16,23,34,35,36]. In particular, all these findings provide additional insight on the potential hypothesis that the SatDNA assay alone appears to be less sensitive. In our study, 14.29% of the samples were confirmed positive with molecular techniques. Half of them (7.14% of the 75 total subjects) gave positive results to the SatDNA target performed by both PCRs, the commercial pmPCR, as well as by the in-house PCR, while the other half of samples were positive only for the kDNA target by ihPCR (Figure 2).

In accordance with our findings, the available data in the literature report that the kDNA is usually chosen as the best target for the follow-up after the treatment and in the case of organ-transplanted patients [17,37]. Our results reported here are very similar to those recently observed in a study conducted in Chile, an endemic country for Chagas disease. The prevalence rate of the PCR targeting kDNA dropped from 84.2% to 7% in the same cohort of individuals before and after an average time of period of 6 and a half years from the treatment [24]. The last scenario of the treated Chilean cohort is more similar to a cohort of migrants from Latin America attending hospitals in a nonendemic reality, such as our Center for Tropical disease in the north of Italy. To the best of our knowledge, there is only one previous study evaluating the performances of ihPCR developed by Ramirez and co-workers in 2015 and of the commercial RealCycler^®^CHAG kit for the molecular detection of *T. cruzi* [20]. In particular, in this study, Abras and co-workers showed a K value of 0.79 (95% CI 0.69 to 0.9) between the two PCR assays [20]. However, in this previous study, only SatDNA was evaluated. On the contrary, in the present study, we measured both k and Sat DNAs. Regarding the concordance between the tests used in our evaluation, it is worth mentioning that the same four samples positive to SatDNA by ihPCR were also positive to kDNA by ihPCR and SatDNA by pmPCR (Figure 2). Indeed, the agreement of kDNA tested by ihPCR and SatDNA tested by both PCRs (ihPCR and pmPCR) observed in our study was 64.11%. Meanwhile, the overall accuracy for the kDNA target was higher than that observed only for the SatDNA target. It is known that the sensitivity of the PCR might be affected by the changes in the copy number of Sat DNA and in the sequence of both Sat and k DNA of the parasites [25,38,39,40,41,42]. These facts highlight the importance of the PCR primers design considering the genetic variability of *T. cruzi* [41,42]. The ihPCR assay consists of a primer sequence for kDNA potentially more sensitive in the detection of this target region compared to Sat DNA. As reported by Qvastrom and co-workers, this assay for kDNA detection is able to also amplify a non-*T. cruzi* DNA (i.e., *T. rangeli*) [32]. All these findings support the use of kDNA combined with SatDNA in order to enhance the sensitivity and specificity of *T. cruzi* DNA detection. Moreover, based on the concordance of both PCRs, in-house and commercial, to detect Sat DNA and the costs of the two PCRs (Table 2), it would be convinient, in a clinical setting especially in low-income countries, to combine Sat and k DNA targets in a multiplex ihPCR. Nevertheless, further assesment of this method should be perfomed and validated.

However, the present study has some limitations: (i) The potential low parasite burden as enrolled individuals were in the chronic phase of the disease; (ii) most of our Chagas disease patients came from Bolivia; thus, our data might not be representative of the general geographic range and diversity of *T. cruzi* (*T. cruzi* I is substantially absent in Bolivia); (iii) the low sample size.

## 5. Conclusions

Overall, our data suggest that the kDNA target might increase the sensitivity of *T. cruzi* detection by a molecular technique. However, due to the possible genetic variability, it could be worth using both the target k and Sat DNAs, as indicated previously [28,34]. The overall sensitivities obtained in this study from both PCR assays are very low, suggesting that this approach of analysis cannot be considered a confirmatory test for clinic patients or blood donors, and the serology is still the first choice. Thus, our data seem to be in agreement with the WHO recommendation, according to which the biomolecular methods cannot be used for Chagas disease screening purposes and should be used as confirmatory methods only under particular conditions (perinatal or congenital transmission and evaluation of treatment follow-up, two testing recommended) [14]. To the best of our knowledge, a common strategy for the molecular detection of *T. cruzi* DNA in blood samples is still not established. Generally, our findings confirm that the best option nowadays to screen patients with chronic Chagas diseases in a nonendemic area are serological assays, as suggested by the WHO. Despite this, our finding suggests that the analysis of both targets k and Sat should be considered when a PCR for Chagas diseases is performed in a nonendemic reality.

In our retrospective study, presented here, *T. cruzi* discrete typing unit (DTU) identification was not possible, due to the limited available samples. Future studies are needed to include a higher number of samples to assess the optimal real-time PCR-based approach for the detection of *T. cruzi* infection, especially in nonendemic countries. Moreover, prospective analysis using a multiplex PCR approach should be performed to provide new insight into the DTU characterization.

## Figures and Tables

**Table 1:** Subjects demographic characteristics.

	CD-EC	N-EC	N-NEC
No. of patients	56	6	13
Origins	Bolivia (55)	Bolivia (6)	Italy (13)
	Brazil (1)		
Sex M/F	18/38	1/5	4/9
Median age in years sex M(IQR)and F (IQR)	38(36–45) and 42(33–49)	21(21–21) and 44(17–47)	39(32–45) and 35(31–45)
Clinical manifestations	Cardiac (5)	None	None
	Digestive (15)		
	Indeterminate (36)		
Serology results	* Positive (56)	Negative (6)	Negative (13)

CD-EC: Chronic Chagas disease from endemic country; N-EC: Negative from endemic country; N-NEC: Negative from nonendemic country; IQR: Interquartile range. * Positive = positive results to at least two out of three serological exams performed.

**Table 2:** Technical characteristics of the in-house PCR and the RealCycler^®^ CHAG kit (Progenie molecular).

	ihPCR	pmPCR
Target	Sat and k regions	Sat region
PCR type	Qualitative real-time PCR	Qualitative real-time PCR
Probe type	TaqMan MGB (SatDNA) LNA probe (kDNA)	Hydrolysis
Internal control used for detection of PCR inhibitors	Yes (*A. thaliana* DNA exogenous internal control)	Yes (Competitive Heterologous Internal Control)
Ready-to-use PCR mix	No	Yes
Cost per single sample	* ≈ 10 €	** ≈ 32 €
Duration of PCR	≈83’	≈93’

ihPCR: In-house PCR [18]; pmPCR: Commercial RealCycler^®^ CHAG Progenie Molecular PCR. * Cost calculated as price, for all the targets analyzed, of consumables and reagents used in the routine procedure of our laboratory; ** Actual price refers to the Italian market.

**Table 3:** Performance of the two PCR assays for the detection of *T. cruzi* DNA when compared to the known Chagas disease (positive to at least two serological tests) diagnosis in 75 blood samples.

		TP	FN	FP	TN	%Se (CI)	%Sp (CI)
ihPCR	(kDNA)	8	48	0	19	14.29 (6.38–26.22)	100 (82.35–100)
	(SatDNA)	4	52	0	19	7.14 (19.8–17.29)	100 (82.35–100)
pmPCR	(SatDNA)	4	52	0	19	7.14 (19.8–17.29)	100 (82.35–100)

TP = number of true positives, FP = number of false positives, TN = number of true negatives, and FN = number of false negatives, Se = sensitivity, Sp = specificity, CI = 95% confidence interval, ihPCR = in-house real-time PCR [28], and pmPCR = commercial RealCycler^®^ CHAG Progenie Molecular PCR.

## Supplementary Materials

The following are available online at https://www.mdpi.com/2076-2607/8/11/1692/s1. The data supporting the findings of this study are included in this published article and its supplementary material (Table S1. Study database).
